# Prioritising the application of genomic medicine

**DOI:** 10.1038/s41525-017-0037-0

**Published:** 2017-11-21

**Authors:** Brett Doble, Deborah J. Schofield, Tony Roscioli, John S. Mattick

**Affiliations:** 1 Garvan Institute of Medical Research, Sydney, NSW 2010 Australia; 2 Health Economics Research Centre, Nuffield Department of Population Health, University of Oxford, Oxford, OX3 7LF UK; 3 Faculty of Pharmacy, The University of Sydney, Sydney, NSW 2006 Australia; 4 Murdoch Childrens Research Institute, Royal Children’s Hospital, Melbourne, VIC 3052 Australia; 5 Department of Medical Genetics, Sydney Children’s Hospital, Sydney, NSW 2031 Australia; 6 St. Vincent’s Clinical School, UNSW Australia, Sydney, NSW 2052 Australia

## Abstract

The clinical translation of genomic sequencing is hampered by the limited information available to guide investment into those areas where genomics is well placed to deliver improved health and economic outcomes. To date, genomic medicine has achieved its greatest successes through applications to diseases that have a high genotype–phenotype correlation and high penetrance, with a near certainty that the individual will develop the condition in the presence of the genotype. It has been anticipated that genomics will play an important role in promoting population health by targeting at-risk individuals and reducing the incidence of highly prevalent, costly, complex diseases, with potential applications across screening, prevention, and treatment decisions. However, where primary or secondary prevention requires behavioural changes, there is currently very little evidence to support reduction in disease incidence. A better understanding of the relationship between genomic variation and complex diseases will be necessary before effective genomic risk identification and management of the risk of complex diseases in healthy individuals can be carried out in clinical practice. Our recommended approach is that priority for genomic testing should focus on diseases where there is strong genotype–phenotype correlation, high or certain penetrance, the effects of the disease are serious and near-term, there is the potential for prevention and/or treatment, and the net costs incurred are acceptable for the health gains achieved.

## Introduction

Genomic sequencing is revolutionising research into human diversity and the causes of disease, resulting in improved diagnoses through the identification of molecular etiologies and the ability to manage many costly and disabling conditions. This is leading to increased investment by governments in genomic medicine, focussing initially on rare diseases and cancer, with the longer-term intent of incorporating genomic information into a new data ecology for biomedical discovery, more precise medical treatment, better stratification of patient populations and more efficient/cost-effective use of healthcare resources.

Currently genomic sequencing has achieved diagnostic rates for individually rare, but collectively common, monogenic diseases as high as in excess of 50%^1^ (with whole genome sequencing (WGS) typically producing a higher diagnostic rate than WES or earlier technologies),^2^ with the potential to reduce lengthy and expensive diagnostic pathways,^3^ as well as generate lifetime health benefits through application of appropriate treatments,^4^ when available. Clinical guidelines for pharmacogenomic testing are also available for a number of gene–drug pairs,^5^ while clinical genomic testing in the context of common complex diseases is more limited, with few robust polygenic risk scores being available.^6^

The clinical translation of genomic sequencing is further hampered by the limited information available to guide investment into those areas where genomics is well placed to deliver improved health and economic outcomes. There is a need for identification of the key elements of a framework for distinguishing applications of genomics that are likely to have demonstrable value and meet current effectiveness and cost-effectiveness evaluation guidelines, by prioritising the most pertinent genomic information to ensure genomic sequencing is most productively and efficiently applied in clinical practice.

## First steps in implementing genomic medicine

Advancements in genomic sequencing enable more accurate diagnoses, rational disease prevention strategies, better treatment selection, and the development of novel therapies. This approach to medical care has been called many different names, such as genomic, personalised or precision medicine (see Table 1), but here we refer to genomic medicine as “the use of genomic information and technologies to determine disease risk and predisposition, diagnosis and prognosis, and the selection and prioritisation of therapeutic options”.^7^

**Table 1:** Nomenclature and definitions

Personalised medicine—“the tailoring of medical treatment to the individual characteristics of each patient”^33^ This term was originally used to describe the shift in focus in clinical decision making to a more individualised approach, largely supported by molecular information. At present the term is still used widely by the media, governments and lay community, but its use has been criticised by experts as it is often misinterpreted as implying unique treatment could be designed for each individual.^34^ Preference is, therefore, now given to alternative terms listed below depending on jurisdiction and context
Precision medicine—“an approach to disease treatment and prevention that seeks to maximise effectiveness by taking into account individual variability in genes, environment, and lifestyle”^35^ Precision medicine can be considered an all-encompassing term that includes more specific components related to the use of specific technologies and/or information, including the three terms listed below
Stratified medicine—“the grouping of patients according to disease risk or likely treatment response, as determined by diagnostic tests, to determine the course of care”^36^ This term is most commonly used within the UK
Genomic medicine—“the use of genomic information and technologies (e.g., genomic sequencing, which includes whole genome and exome sequencing and multi-gene panels) to determine disease risk and predisposition, diagnosis and prognosis, and the selection and prioritisation of therapeutic options”^5^ This term is the focus of this paper and has a limited focus on genomic information as opposed to consideration of variability in other factors such as environment and lifestyle
Genomic sequencing—includes the use of whole genome sequencing, whole exome sequencing and gene panels
Pharmacogenomics—“a polygenic or genome-wide approach to identifying genetic determinants of drug response, capitalising on information from the Human Genome Project and on advances in technology”^37^

When individuals with a condition with high penetrance (with near certainty the individual will develop the condition) are identified, there is a greater likelihood of preventative or mitigating interventions being successful. Accordingly, genomic medicine has achieved its greatest successes through applications to high penetrance monogenic diseases. Significant health benefits from the provision of genomic information using this criterion are already demonstrable, even when the behavioural modification required is arduous.

There are, as yet, few examples of the use of genomic sequencing with accompanying costs of the impact of change in disease trajectory, thus we will draw lessons from available studies, including in some cases conditions identified with older technologies such as biochemical or single gene tests.

Phenylketonuria (PKU) is autosomal recessive disorder, caused by mutations in both alleles of the gene for phenylalanine hydroxylase. Although it is more cheaply diagnosed with a biochemical test, the management of this inborn error of metabolism is an important example of substantial therapeutic benefit being successfully achieved as a result of significant behavioural modification following the provision of genetic information. Delay in dietary treatment can have significant impacts on a child’s intellectual development. Maintaining a phenylalanine-restricted diet is quite onerous, yet despite this burden, the incidence of the cognitive impacts of PKU has been markedly reduced (by 92%) as a result of newborn screening and dietary advice^8^ (Table 2). Genomic testing in the context of PKU is most likely to be most relevant as either a prenatal or preconception carrier screening test to restore reproductive confidence, however, this would also necessitate identifying the disease-causing mutation in the affected child first using genomic testing.

**Table 2:** Reduction of the incidence of expensive diseases with genetic information

Disease	Incidence	Intervention	Reduction in incidence^a,b^	Lifetime per patient direct medical costs of disease (USD 2016)^c,d^	Annual direct medical costs of disease incurred by health system (USD 2016)^c,e,f^
Phenylketonuria (PKU)	1/10,000^38^	Dietary treatment with a phenylalanine restricted diet	92%^6^	$32,930/patient (up to 36 years of life)^39^	—
β-thalassemia major	1/100,000^38^	Reproductive planning	90%^10^	$1,370,182/patient (60 year lifespan)^40^	—
Cystic fibrosis (CF)	1/8,000 to 1/10,000^38^	Reproductive planning	65%^9^	$511,961/patient (28 year lifespan)^41^	—
Fragile X syndrome (FXS)	1/4,000 to 1/5,000^38^	Reproductive planning	47%^8^	$679,469/patient (72 year lifespan)^42^	—
Familial hypercholesterolaemia (FH)	1/500 for heterozygous dominantly inherited; 1/1,000,000 for homozygous autosomal recessive inherited^38^	Prophylactic statin therapy	51%^7^	—	$2,931,345,622^43^ (per annum)
Adverse drug reactions (ADRs)	2,216,000 hospitalised patients in the US had serious ADRs; 106,000 had fatal ADRs^44^ 1/16 hospitalisations in the UK are a result of ADRs^45^	Informed prescribing	17% (4–30%)^17^	—	$242,392,437,330^46^ (per annum)

^a^ For PKU percentage refers to the reduction in PKU patients with low IQ (<90)

^b^ For FH percentage refers to the reduction in major adverse cardiovascular events in patients with homozygous FH after receiving lipid-lowering therapy

^c^ All costs were inflated to 2016 prices using country-specific (United Kingdom or United States) OECD all items non-food, non-energy Consumer Price Indexes (http://stats.oecd.org/Index.aspx?DatasetCode=MEI_PRICES) and converted to United States dollars (1 GBP = 1.29 USD) as to report all costs in a common currency and year

^d^ For PKU the cost refers to the per-patient lifetime direct medical costs of a PKU patient that does not receive a phenylalanine restricted diet, thereby representing the direct medical costs of not identifying an individual with PKU

^e^ For FH the cost refers to the annual medical costs of coronary heart disease across all of the United Kingdom, which highlights the magnitude of the direct medical costs that are associated with the condition. The argument being, if prophylactic statin therapy were used in all individuals identified to have either heterozygous or homozygous FH through genomic sequencing, a 51% reduction in the incidence of major cardiovascular events would therefore result in large savings to health care systems in terms of direct medical costs

^f^ For ADRs the cost refers to the annual medical costs of drug-related morbidity and mortality in the United States, which highlights the magnitude of the direct medical costs that are associated with ADRs. Even a modest reduction of 17% in the incidence of these events through more informed prescribing after genomic testing would therefore result in large savings to health care systems in terms of direct medical costs

Molecular testing can also have an immediate and effective application to other common monogenic diseases that meet our recommended criteria of high penetrance and significant health effects (see below). Identifying patients with familial hypercholesterolaemia via genetic testing allows for the application of prophylactic therapy (statins), which has resulted in a reduction of death and major adverse cardiovascular events of 66 and 51%, respectively.^9^ Similarly, a reduction in the incidence of serious and costly monogenic conditions such as fragile X syndrome, cystic fibrosis, and β-thalassaemia of 47–90%^10–12^ has been reported as a result of informed family planning following preconception genetic testing (Table 2), with the added benefit of restored reproductive confidence. This is a remarkable step forward from the time when a majority of families, even those with a family history (FH) of a serious genetic disorder, did not have a molecular diagnosis to inform reproductive management. With genomic testing, many more families could obtain an accurate assessment of their risk of having a child with a severe genetic disorder, and have their reproductive confidence restored through preconception carrier screening and preimplantation genetic diagnosis or invasive testing in pregnancy.

Additional examples demonstrate an emerging capacity to obtain a molecular diagnosis and offer treatment particularly in rare childhood diseases as new causal genes (e.g., there are more than 1000 monogenic causes of intellectual disability now known) are identified.^13^ New therapies are emerging such as a study reporting on 81 inborn errors of metabolism, with therapies including diet, co-factor/vitamin supplements, small molecule substrate inhibition, bone marrow and hematopoietic stem cell transplantation and gene therapy.^14^ With the exception of gene therapy and stem cell transplantation, these treatments may be relatively accessible and affordable. While there are no published studies yet of the long-term impact in terms of a molecular diagnosis on health outcomes and cost of change of management, there are a small number of published studies in clinical cohorts demonstrating that the diagnostic costs are lower when using genomic sequencing in childhood syndromes and neuromuscular disorders such as Stark et al.,^3^ Tan et al.^15^ and Schofield et al.^16^ and while another by Tsiplova et al. reported similar findings for autism spectrum disorder, but using a hypothetical cohort.^17^ Sagoo et al. on the other hand reported a higher diagnostic rate, but at higher cost for a series of cohorts.^18^

There is also potential for genomic sequencing to reduce the significant economic burden associated with adverse drug reactions (Table 2), which can potentially be reduced by 4–30%.^19^ The annual cost of medical care associated with this group of preventable conditions is so high that the implementation of one genomic test early in life may be a cost-effective use of limited healthcare resources (Table 2).^19^

In cancer too we are beginning to see evidence of cost-effective interventions particularly in relation to screening, where the cost of prophylactic screening is much lower than treatment of the cancer itself. For example, Gallego et al.^20^ reported screening for Lynch syndrome using next generation sequencing panels was cost effective, while Li et al.^21^ reported screening women at risk of hereditary breast cancer with prophylactic intervention was similarly cost-effective.

Thus, we argue, the priority for genomic testing should be to identify individuals at high risk of imminent, serious, preventable (or reversible) disorders that are cost effective to treat. These patients and their families are well placed to benefit from genomic medicine through sequencing of affected children and provision of targeted therapies where available. There are already population-specific screening programmes demonstrated to be effective in identifying parents at high risk of having children with such disorders,^12^ but there is a need to assess the feasibility and cost-effectiveness of expanded genomic carrier screening. Existing genomic medicine and carrier screening programmes are not universally available. They are often still within a research context, which in time, will provide evidence on incremental health gains and cost-effectiveness, which is valuable for making the case for universal access and public funding.

## Expanding genomic medicine to complex diseases

There has been considerable hope for the successful application of genomic medicine to common complex diseases, such as heart disease, cancer, obesity, diabetes and lung disease. It has been suggested that adding personal genetic risk (usually based on a combination of low penetrance alleles) to general risk information (i.e., lifestyle factors, medical and FH) could impact on individual behaviour and potentially prevent such diseases from developing, however evidence for the utility of genomic testing in this context is not yet available.

However, the evidence from an updated Cochrane review and meta-analysis^22^ provides little support for behavioural change when healthy individuals are presented with genetic information compared to general risk information concerning their risk of various complex diseases. Since the publication of the Cochrane review in 2016 evidence of behavioural change has been noted in individual studies, but generally the impact on disease incidence is small. For example, one study indicated that the use of statins with the provision of both genetic and general risk information for coronary heart disease (CHD), compared to only general risk information alone, would result in a reduction in CHD incidence over 10-years in a high genetic risk population of only ~5%,^23^ assuming a ~45% relative-risk reduction from high-intensity statin therapy.^24^

In another recent study, individuals receiving genetic risk information for Alzheimer’s disease and coronary artery disease together (compared to only receiving genetic risk information for Alzheimer’s disease alone) reported more health behaviour changes related to diet, exercise, medications, dietary supplements and stress reduction.^25^ This one study alone indicates a greater likelihood of behavioural change when multiple genetic risks are presented together and at least one of the disease risks are modifiable through medical intervention. Although this study did not report long enough follow-up to determine whether the intervention reduced Alzheimer’s disease or coronary artery disease incidence and does not supersede the negative effect noted in the majority of the evidence synthesised in the updated Cochrane review.^22^ Similarly, Vassy et al. report on the use of WGS plus FH over FH alone in primary care for health adult patients with new clinical actions in 34% of the WGS plus FH patients compared 16% for FH alone. Only 2 out of the 11 patients with an identified Mendelian allele manifested a phenotype consistent with at least 80% non-penetrance. The study was also limited by a small sample size (100 patients) with no follow-up data reporting on whether patients obtained a significant health benefit.^26^

Currently there is limited clinical and economic evidence of the utility of genomic sequencing in common complex diseases, and of those studies reported some have significant limitations. For example, Dzau et al. estimated that reporting genomic variants in these diseases would lead to modulation of health-related behaviours with a 10–50% reduction in disease incidence, valued at $US33–607 (£22–405) billion per condition.^27^ However, this estimate was predicated on the assumption that the provision of genomic information will overcome the limitations of current lifestyle interventions where adherence to such programmes has traditionally been poor on a population level. As outlined above, the evidence does not support this assumption, and thus the projected benefits are in all likelihood vastly overestimated.

This is not to say that genomics is not relevant to the reduction of complex diseases, but rather it is necessary to better understand the genotype–phenotype relationship in complex diseases before effective genomic risk identification and management of complex diseases can be implemented in clinical practice.

Furthermore, some conditions traditionally classified as complex diseases are in fact disease clusters with many causes. For example, there are nearly 200 genetic variants known to have an effect on blood cholesterol, which may, in part, impact response to lipid lowering medication.^28^ As an example, in some populations receiving statins, only a small proportion of patients achieve target reductions in plasma cholesterol levels.^29^ Thus, targeting group health risk modification at a large cluster of conditions categorised under a single heading may obscure the potential of genomic medicine in complex diseases. Applying genomic medicine to stratify and target specific interventions to individual genetic variation, and along with better knowledge of the genotype–phenotype correlations will enable better outcomes, both in therapeutic and behavioural responses.

## A framework for distinguishing applications of genomics likely to have demonstrable value in reducing disease burden and costs of disease

**Fig. 1:**
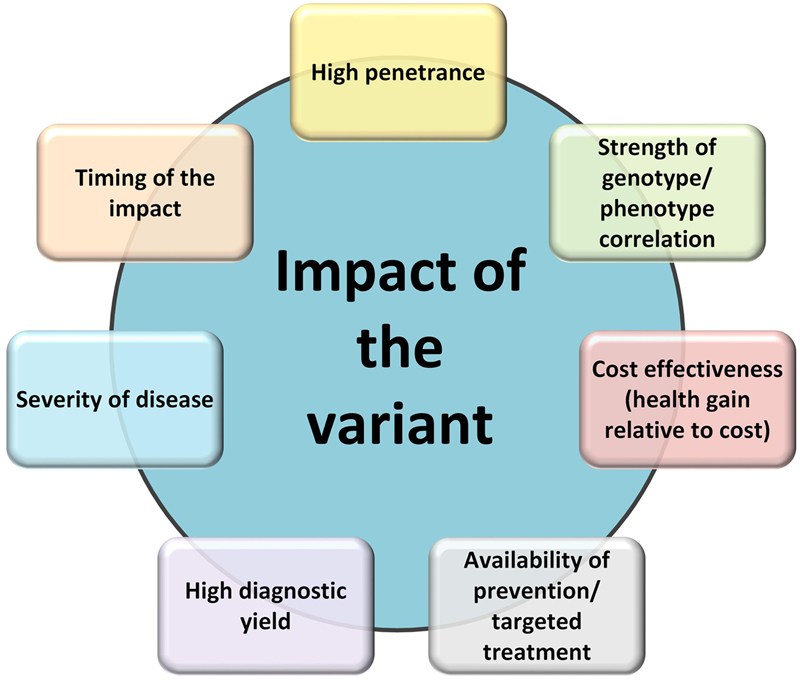
Framework for distinguishing applications of genomics likely to have demonstrable value

While there are well established guidelines for the conduct of an evaluation of clinical and cost-effectiveness within medicine such as those from the National Institute for Health and Clinical Excellence,^30^ as well as more recent frameworks for the evaluation of genomics,^31^ there is little to guide researchers on which areas of medicine are likely to yield outcomes that might meet the guidelines. The evidence to date suggests that based on our current knowledge, there is greater potential for deriving value from genomic sequencing (with its capacity to test many conditions simultaneously) when applied to monogenic disorders, with enormous potential to improve health outcomes through screening,^8,10–12^ disease prevention and change of management. Our synthesis of the examples included in this paper suggest that the distinguishing characteristics of applications of genomics with significant capacity to reduce the incidence of costly illness (Fig. 1) are the:

- strength of the genotype–phenotype correlation,
- high penetrance,
- imminence of severe illness,
- severity of the disease impact,
- relatively high diagnostic yield,
- availability of prevention or targeted treatment, and
- the net costs incurred are acceptable for the health gains achieved.

Promising areas that with further evidence in relation to health outcomes and/or cost impacts meet all or most of the criteria set out in our framework include: inborn errors of metabolism,^14^ neuromuscular disorders,^16^ neurodevelopment disorders,^3,32^ inherited cancer risk^20,21^ preconception carrier screening or prenatal screening^12^ and in the paediatric and neonatal intensive care unit.^33^ The importance of critical criterion in our framework such as diagnostic rate, health outcomes due to prevention or therapy and health economic outcomes are highlighted in a paper by Gaff et al.^34^

The urgent need for evidence related to cost-effectiveness that takes account of both diagnostic rate and health outcomes as described in our framework is highlighted by Sagoo et al.: the main limitation of the studies to date is that while it is possible to derive an incremental cost-effectiveness ratio from a relative diagnostic rate and cost for genomic sequencing, there is “no universally acknowledged willingness-to-pay threshold for a diagnosis”, and this makes an incremental cost per additional diagnosis difficult to interpret.^18^

## Improving access and usability of genomic databases

While existing databases such as OMIM^®^ provide a wealth of descriptive information concerning genomic variants, they do not allow the effect of a specific genomic variant to be predicted. In contrast, the ClinVar database provides specific variant information that predicts the relationship between human genomic variation and expressed phenotypes using supporting evidence. These are, however, limited in their scope, in that, ClinVar is currently largely incomplete and the ACMG list is limited to only genes that should be reported as additional findings rather than variants that might be of interest in the primary use of testing.^35^ To improve accessibility of genomic information, there is a need for comprehensive, evidence-based, continuously curated, user-friendly genotype–phenotype databases to aid diagnosis and prognosis, and thereby appropriate treatment and prevention to capitalise on the promise of genomic medicine. Applying genomics to conditions that meet one of our main criterion for prioritisation of receiving genomic testing (i.e., a strong genotype–phenotype relationship) reduces the potential for false positives, while continuous curation is important in reducing the potential for both false positives and false negatives. With this information readily available from such databases in the future, numerous rare but collectively common and very costly diseases could be prevented (such as those listed in Table 2 and others fitting our recommended criteria), and treating patients with expensive but ineffective treatments could be avoided, offering large cost savings that create headroom to treat other patients with effective targeted interventions.

## Fulfilling the promise of genomic medicine

We are in a period of tremendous innovation in genomic medicine and large population studies such as Genomics England’s 100,000 Genomes Project and the All of Us programme in the United States hold great promise in identifying further conditions where highly penetrant variants causing serious disease might be much more effectively treated. To facilitate rapid translation of evidence based genomic medicine, there will also need to be increased capacity within the health system, particularly in laboratory and genetic services. The evidence suggests that deriving value from genomic medicine, in the short term at least, will be a function of the strength of the genotype–phenotype correlation (high penetrance), the severity of the disease impact, the availability of prevention or targeted treatment, and the net costs incurred for the health gains achieved. Further, widely available comprehensive, evidence-based, continuously curated, user-friendly genotype–phenotype databases of genomic approaches to treatment and prevention, which will be supported by data collected in the large population studies currently ongoing, will maximise the benefits from genomic medicine.

## References

[1] Technology Evaluation Center. *Special Report: Exome Sequencing for Clinical Diagnosis of Patients with Suspected Genetic Disorders* Vol. 28 (BlueCross BlueShield Association, Chicago, 2015). 24066368

[2] Lionel AC (2017). Improved diagnostic yield compared with targeted gene sequencing panels suggests a role for whole-genome sequencing as a first-tier genetic test. Genet. Med.

[3] Stark Z (2017). Prospective comparison of the cost-effectiveness of clinical whole-exome sequencing with that of usual care overwhelmingly supports early use and reimbursement. Genet. Med.

[4] Stavropoulos, D. J. et al. Whole-genome sequencing expands diagnostic utility and improves clinical management in paediatric medicine. *npj Genomic Medicine* **1**, 15012 (2016). 10.1038/npjgenmed.2015.12 PMC5447450 28567303

[5] Caudle KE (2014). Incorporation of pharmacogenomics into routine clinical practice: the clinical pharmacogenetics implementation consortium (CPIC) guideline development process. Curr. Drug. Metab.

[6] Chatterjee N, Shi J, Garcia-Closas M (2016). Developing and evaluating polygenic risk prediction models for stratified disease prevention. Nat. Rev. Genet.

[7] Science and Technology Committee—House of Lords. *Genomic Medicine, 2nd Report of Session 2008–09 Volume I: Report* (Authority of the House of Lords, London, 2009).

[8] The Advisory Committee on Inborn Errors of Metabolism to the Ministry of Health (1973). PKU screening — Is it worth it?. Can. Med. Assoc. J.

[9] Raal FJ (2011). Reduction in mortality in subjects with homozygous familial hypercholesterolemia associated with advances in lipid-lowering therapy. Circulation.

[10] Turner G (2008). Restoring reproductive confidence in families with X-linked mental retardation by finding the causal mutation. Clin. Genet.

[11] Cunningham S, Marshall T (1998). Influence of five years of antenatal screening on the paediatric cystic fibrosis population in one region. Arch. Dis. Child.

[12] Zlotogora J (2009). Population programs for the detection of couples at risk for severe monogenic genetic diseases. Hum. Genet.

[13] Vissers LE, Gilissen C, Veltman JA (2016). Genetic studies in intellectual disability and related disorders. Nat. Rev. Genet.

[14] van Karnebeek CD, Stockler S (2012). Treatable inborn errors of metabolism causing intellectual disability: a systematic literature review. Mol. Genet. Metab.

[15] Tan TY (2017). Diagnostic Impact and Cost-effectiveness of Whole-Exome Sequencing for Ambulant Children With Suspected Monogenic Conditions. JAMA pediatrics.

[16] Schofield D (2017). Cost-effectiveness of massively parallel sequencing for diagnosis of paediatric muscle diseases. npj Genomic Medicine.

[17] Tsiplova K (2017). A microcosting and cost-consequence analysis of clinical genomic testing strategies in autism spectrum disorder. Genet. Med.

[18] Sagoo, G. S., Norbury, G., Mohammed, S. & Kroese, M. *The budget impact and cost-effectiveness of introducing whole-exome sequencing-based virtual gene panel tests into routine clinical genetics* (PHG Foundation, Cambridge, 2017).

[19] Alagoz O, Durham D, Kasirajan K (2016). Cost-effectiveness of one-time genetic testing to minimize lifetime adverse drug reactions. Pharmacogenomics. J.

[20] Gallego CJ (2015). Next-generation sequencing panels for the diagnosis of colorectal cancer and polyposis syndromes: a cost-effectiveness analysis. J. Clin. Oncol.

[21] Li Y (2017). A Multigene Test Could Cost-Effectively Help Extend Life Expectancy for Women at Risk of Hereditary Breast Cancer. Value Health.

[22] Hollands GJ (2016). The impact of communicating genetic risks of disease on risk-reducing health behaviour: systematic review with meta-analysis. BMJ.

[23] Kullo IJ (2016). Incorporating a genetic risk score into coronary heart disease Risk estimates: effect on LDL cholesterol levels (the MIGENES clinical trial). Circulation.

[24] Stone NJ (2014). 2013 ACC/AHA guideline on the treatment of blood cholesterol to reduce atherosclerotic cardiovascular risk in adults: a report of the American College of Cardiology/American Heart Association Task Force on Practice Guidelines. J. Am. Coll. Cardiol.

[25] Christensen KD (2016). Disclosing pleiotropic effects during genetic risk assessment for alzheimer disease: a randomized trial. Ann. Intern. Med.

[26] Vassy JL (2017). The impact of whole-genome sequencing on the primary care and outcomes of healthy adult patients: a pilot randomized trial. Ann. Intern. Med.

[27] Dzau VJ, Ginsburg GS, Van Nuys K, Agus D, Goldman D (2015). Aligning incentives to fulfil the promise of personalised medicine. Lancet.

[28] Surakka I (2015). The impact of low-frequency and rare variants on lipid levels. Nat. Genet.

[29] Pijlman AH (2010). Evaluation of cholesterol lowering treatment of patients with familial hypercholesterolemia: a large cross-sectional study in The Netherlands. Atherosclerosis.

[30] National Institute for Health and Care Excellence (NICE). *Guide to the Methods of Technology Appraisal 2013* (NICE, London, 2013). 27905712

[31] Canadian Agency for Drugs and Technologies in Health (CADTH). *Evaluation Frameworks for Genetic Tests: Environmental Scan, Issue 37* (CADTH, Ottawa, 2012).

[32] Soden SE (2014). Effectiveness of exome and genome sequencing guided by acuity of illness for diagnosis of neurodevelopmental disorders. Sci. Transl. Med.

[33] Willig LK (2015). Whole-genome sequencing for identification of Mendelian disorders in critically ill infants: a retrospective analysis of diagnostic and clinical findings. Lancet Resp. Med.

[34] Gaff CL (2017). Preparing for genomic medicine: a real world demonstration of health system change. npj Genomic Medicine.

[35] ACMG Board of Directors. ACMG policy statement: updated recommendations regarding analysis and reporting of secondary findings in clinical genome-scale sequencing. *Genet. Med*. **17**, 68–69, 10.1038/gim.2014.151 (2015). 10.1038/gim.2014.151 25356965

[36] The Academy of Medical Sciences. Realising the potential of stratified medicine. London, UK: The Academy of Medical Sciences, July 2013.

[37] Evans WE, Relling MV (2004). Moving towards individualized medicine with pharmacogenomics. Nature.

[38] Orphanet: an online database of rare diseases and orphan drugs. (INSERM, 1997)

[39] Guest JF, Bai JJ, Taylor RR (2013). Costs and outcomes over 36 years of patients with phenylketonuria who do and do not remain on a phenylalanine-restricted diet. Journal of Intellectual Disability Research.

[40] Karnon J, Zeuner D, Brown J (1999). Lifetime treatment costs of β-thalassaemia major. Clinical & Laboratory Haematology.

[41] U.S. Congress Office of Technology Assessment. Cystic Fibrosis and DNA Tests: Implications of Carrier Screening, OTA-BA-532. Washington DC: U.S. Government Printing Office, August 1992.

[42] Sacco P, Capkun-Niggli G, Zhang X (2013). The economic burden of fragile x syndrome: healthcare resource utilization in the United States. American health & drug benefits.

[43] Liu JLY, Maniadakis N, Gray A (2002). The economic burden of coronary heart disease in the UK. Heart.

[44] Lazarou J, Pomeranz BH, Corey PN (1998). Incidence of adverse drug reactions in hospitalized patients: A meta-analysis of prospective studies. JAMA.

[45] Pirmohamed M, James S, Meakin S (2004). Adverse drug reactions as cause of admission to hospital: prospective analysis of 18 820 patients. BMJ: British Medical Journal.

[46] Ernst, F. R. & Grizzle, A. J. Drug-related morbidity and mortality: updating the cost-of-illness model. *Journal of the American Pharmaceutical Association* **41**, 192–9 (2001). 10.1016/s1086-5802(16)31229-3 11297331

